# From ECG signals to images: a transformation based approach for deep learning

**DOI:** 10.7717/peerj-cs.386

**Published:** 2021-02-10

**Authors:** Mahwish Naz, Jamal Hussain Shah, Muhammad Attique Khan, Muhammad Sharif, Mudassar Raza, Robertas Damaševičius

**Affiliations:** 1 COMSATS University Islamabad, Wah, Pakistan; 2 HITEC University, Taxila, Pakistan; 3 Department of Applied Informatics, Vytautas Magnus University, Kaunas, Lithuania

**Keywords:** ECG, Deep features, Image processing, Deep learning, Convolutional neural networks, Feature fusion

## Abstract

Provocative heart disease is related to ventricular arrhythmias (VA). Ventricular tachyarrhythmia is an irregular and fast heart rhythm that emerges from inappropriate electrical impulses in the ventricles of the heart. Different types of arrhythmias are associated with different patterns, which can be identified. An electrocardiogram (ECG) is the major analytical tool used to interpret and record ECG signals. ECG signals are nonlinear and difficult to interpret and analyze. We propose a new deep learning approach for the detection of VA. Initially, the ECG signals are transformed into images that have not been done before. Later, these images are normalized and utilized to train the AlexNet, VGG-16 and Inception-v3 deep learning models. Transfer learning is performed to train a model and extract the deep features from different output layers. After that, the features are fused by a concatenation approach, and the best features are selected using a heuristic entropy calculation approach. Finally, supervised learning classifiers are utilized for final feature classification. The results are evaluated on the MIT-BIH dataset and achieved an accuracy of 97.6% (using Cubic Support Vector Machine as a final stage classifier).

## Introduction

In 2015, according to the United Nations report, the world is facing an aging population. The number of people aged 60 years or more will rise to 56.00% by 2030 or double by 2050 (Escobar, 2011). One of the main fatalities throughout the world is cardiovascular ailments. The human cardiovascular system weakens as we grow older and it is more likely to suffer from arrhythmias. A ventricular arrhythmia is an irregular heartbeat of ventricular rhythm. If not treated in time, it can cause life in danger. Ventricular fibrillation (Vfib), atrial fibrillation (Afib) and atrial flutter (Afl) are the recurrent dangerous arrhythmias that can disturb the aging population (Van Huls Van Taxis, 2019). Ventricular arrhythmias (VA) reduces ventricular function. It may cause the need for implanting a fixed cardioverter defibrillator due to the occurrence of VA during long-standing follow up in patients affected with hypothetical myocarditis (Sharma et al., 2019b).

Different types of arrhythmias are associated with different heartbeat patterns. It is possible to identify these patterns and their types. An electrocardiogram (ECG) is the prime diagnostic tool that works to interpret ECG signals. ECG is a non-invasive recording by skin electrodes that is processed by an ECG device. An ECG shows a voltage between electrode pairs and the muscle activities of the heart that are measured from different directions (Bosznai, Ender & Sántha, 2009). The ECG is an analytic apparatus that processes the electrical action and records the actions of the heart. Interpretation of these subtleties permits determination in a comprehensive scope of heart ailments. These heart ailments can differ from insignificant to hazardous (Elola et al., 2019). To thoroughly see how an ECG uncovers essential data about the state of your heart requires a fundamental comprehension of the life systems and physiology of the heart (Cunningham et al., 2016; Ionasec et al., 2016; Izci et al., 2019). These different kinds of arrhythmias can further be categorized into two major categories. The first one is a single irregular heartbeat, formed arrhythmias, which are called morphological arrhythmias. The second one forms by a set of irregular arrhythmias (Luz et al., 2016).

**Figure 1:**
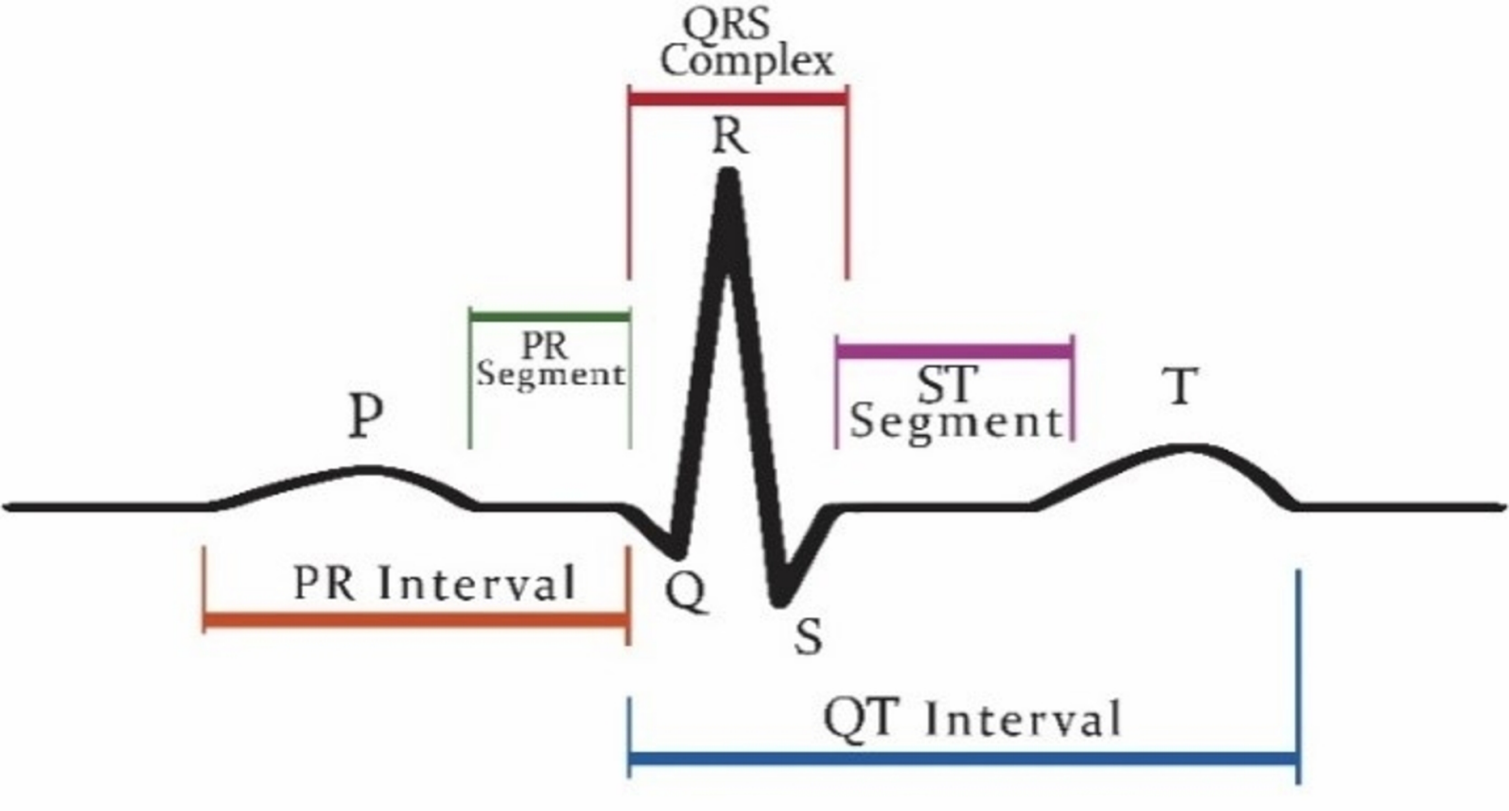
Graphical representation of QRS complex in ECG.

Patients who are suffering from cardiac disease need intervention immediately. For this automated recognition of unusual heartbeats, translation by ECG signals is fundamental. The manual evaluation of these signals is time-consuming and tedious (Acharya et al., 2018). According to new research, (Nigussie & Tadele, 2019) heart attacks and ventricular tachyarrhythmia (VTA) once categorized as “old man’s disease” are now gradually occurring in younger people, especially in women. These irregular rhythms can cause damage to the heart muscle from cardiomyopathy. Now, the major issue is that as we grow older, the human cardiovascular system is more receptive to diseases and becomes weaker (Krbcová et al., 2016). Vfib and VTA are the major arrhythmias reported in the elders (Chow, Marine & Fleg, 2012). While cardiologists can recognize distinctive heartbeat morphologies precisely among various patients, the manual assessment is repetitive and tedious (Srinivas, Basil & Mohan, 2015). The standard deferral between the atria and ventricles contraction of the heart is 0.12–0.20 s. This deferral is superbly coordinated to represent the physical path of the blood from the upper chamber to the ventricle. Intervals can be longer or shorter than this range show potential issues (Madhavapeddi, Verrier & Belardinelli, 2018; Rabey, Cohen & Belhassen, 2018; Sharma et al., 2019a). Figure 1 shows a visualization of the QRS complex.

The most dangerous rhythm is a type of polymorphic ventricular tachycardia (VT) called Vfib (Ibtehaz, Rahman & Rahman, 2019). There are many techniques for the detection of VTA. The most common is the modified Karhunen–Loeve transform, which has been done using a pattern recognition method. Prediction of arrhythmias by applying pattern recognition techniques on ECG data is an emerging and important task in biomedical engineering (Mishra, Arora & Vora, 2019). However, it requires continuous observation of a patient using, for example, wearable sensors (Girčys et al., 2020). Cardiovascular cycle elements reflect basic physiological changes that could predict arrhythmias; however, are obscured by high complexity, no stationary and large inter-individual differences (Sabherwal, Agrawal & Singh, 2019).

Recently, deep learning shows huge success in the medical domain (Anjum et al., 2020). It is used for many imaging (Khan et al., 2020a, 2020b; Fernandes, Rajinikanth & Kadry, 2019), disease recognition (Sahlol et al., 2020a, 2020b; Capizzi et al., 2020), analysis of biomedical signals (Bakiya et al., 2020), Internet-of-Things domain (Huifeng, Kadry & Raj, 2020; Muthu et al., 2020) and epidemic disease spread forecasting (Wieczorek., Siłka & Woźniak, 2020; Wieczorek et al., 2020) tasks.

This paper introduces a new approach to predict VTA and classify various arrhythmias using a novel technique. In this technique, we transform ECG signals into binary images. Our approach differs from other approaches know from the literature as commonly ECG signals are transformed into series data. As a result, deep learning models such as convolution al neural network (CNN) does not work properly on ECG signals data because the minor value of signals data is ignored in the QRS complex thus preventing from accurate recognition of arrhythmias. It is a big challenge to convert the serial signals data into images and further proceed for the detection of VTA.

The novelty and contribution of the article are as follows:

- A novel approach to convert ECG signals into $32 \times 32$ binary images.
- A fusion of features from several deep CNNs for VTA recognition.
- The entropy-based feature selection is employed for obtaining the best feature subset.
- The selected features are finally trained using different classifiers, and higher accuracy is attained as compared to the existing method.

Here are the key advantages that are achieved using our proposed methods:

- No need for complex pre-processing of ECG signals.
- No need for the QRS complex detection.
- Higher accuracy than previous CNN based arrythmia detection techniques.
- Less time consumption for arrythmia detection.

## Related work

Recently, several review articles have been written in this domain which explores the importance of VTA using Deep CNN models. Martis et al. (2014) present three-class learning to inevitably identify Afl, Ventricular flutter (Vfl), normal sinus rhythm (Nsr) and VTA ECG signals. They present effective higher-order bands method on 641,855 and 877(Afib, Nsr and Afl) beats of ECG signals. Formerly, these beats of ECG are exposed to self-governing constituent analysis for the selection of substantial features. The method produced an accuracy of 97.65%, a specificity of 98.75%, and a sensitivity of 98.15% using the k-Nearest Neighbor (KNN) classifier. Acharya et al. (2016) proposed a Computer-aided diagnosis system to automated perceive and classify similar ECG into four classes. These classes are Nsr, Afib and Vfib. They used the complete database acquired from the MIT-BIH arrhythmias database, used entropy features and applied reduction and feature selection from the ECG signals by utilizing a decision tree classifier. This technique achieved accuracy, specificity, and sensitivity of 96.30%, 84.10% and 99.30% respectively. Sufi & Khalil (2010) introduced a data extracting technique with expansion base grouping on compacted ECG signals acquired from the publicly available database. They used features that are correlated by the subset selection technique to decrease them in number. Then the designated features were given to the classifier. The approach predicted Afib, pre-sustained premature ventricular tightening and Vfib and attained an accuracy of 97.00% by using the directive-based system. Wang et al. (2001) presented a unique method for VTA detection. The capability of their technique for clinical utilization and continuous identification was observed utilizing 180 ECG records including Afib, Vfib and Ventricular tachycardia (VT). This technique accomplishes a precision of 97%. Zihlmann, Perekrestenko & Tschannen (2017) presented a convolutional neural system strategy that combined convolutional layers for feature extraction with long-short term memory layers for feature aggregation to recognize the different ECG sections. In their work, they have utilized 5- and 2-s windows of ECG signals without QRS discovery, achieving and *F*-score of 82.1%. Acharya et al. (2016) utilize ECG signal beats; they presented a framework for the automated analysis of certain arrhythmias. They accomplish an accuracy, sensitivity, and specificity of 92.50%, 98.09% and 93.13% individually for the 2-s windows of ECG signals.

The findings of the related literature analysis show that it will be better if we can transform our signals data into images and then merge signal processing with image processing techniques using deep learning. As a result, CNN works better and gets higher accuracy using different classifiers.

## Proposed methodology

### Data used

The signals of ECG were attained from publicly available arrhythmia databases like MIT-BIH, CUDB (Creighton University VT Database) and Nsr. The signals, which are acquired from the MIT-BIH dataset, were recognized and taken out regarding the annotation file, which is set up by the cardiologists (Moody & Mark, 2001). In this work, two lead ECG signals are used. The details of the datasets are given in Table 1. The MIT Arrhythmias dataset consists of signals and their annotated files. The signals data contains a series set of data of each patient with a complete set of ECG patterns of 24 h/s of 36 patients. Each patient’s data has approximately 127,232 series points.

**Table 1:** Publicly available databases.

Database	Taken from
VT (1406072) V_fib_	MIT-BIH Ventricular (VT) MIT-BIH Ventricular Fibrillation(V_fib_)
Nsr (127685) V_fl_	MIT-BIH Arrhythmias (mit db) MIT-BIH Arrhythmias (mit db)
V_fib_ (148654) VT	Creighton university Ventricular Tachyarrhythmia’s (cudb)

### Transformation of signals into images

The proposed VTA detection technique consists of two phases. In the first step, the signal data is transformed into binary images. It is a challenge to convert the serial signals data into images and then proceed further for the detection of VTA. The following are the reasons for adopting the computer vision approach.

- To automate the algorithm of VTA detection using deep CNN.
- To eliminate the need for ECG signal pre-processing.
- The 1D CNN is not working well as compared to 2D CNN on signal data (Wu et al., 2018), therefore there is a need to transform our signals data into images.
- The main problem occurs while findings QRS complex in ECG data. For CNN, there is no need for finding the QRS complex.
- To increase the accuracy and specificity of the approach.

In the second step, deep features are obtained from images. Finally, these extracted features are fused, and selection based on entropy is applied. The selected features are later fed to Support Vector Machine (SVM) and KNN classifiers for classification results.

The following are the phases of transformation signals points into binary images.

### Data normalization

Data normalization is an essential step to VTA detection. Before the transformation, the data must be normalized. Normalization depends on two phases: first, signal data points are split into equal parts which are divisible into total signals points without any data loss. In the second phase, these signals are reshaped into $32 \times 32$ binary images.

Each patient has 24hrs of recorded ECG data, in which we have 127,356 data values. After carefully examining the last value of signals which is repetitive from S to T peak, we subtract the last 380 data values of every patient’s signals data, which are not playing any role in the arrythmia detection, thus obtaining data 126,976 values. To perform transformation, first, we split each data series into 124 segments. Then, every person’s data contain $124 \times 1024$ sequences. Second, we reshape each 1,024-sized segment into an image of $32 \times 32$ size. The result is 124 images for each patient.

Mathematically, we can describe the transformation as an inverse of the vectorization operation, which converts the matrix into a column vector. Specifically, the vectorization of a $m\; \times \; n$ matrix $A$, denoted ${ve{c_{m,n}}}\left( A \right)$, is the $\rm mn\; \times \; 1$ column vector obtained by stacking the columns of $A$ as follows:

${\rm{ve}}{{\rm{c}}_{{\rm{m}},{\rm{n}}}}\left( A \right) = {\left[ {{a_{1,1}},...,{a_{{\rm{m}},1}},{a_{1,2}},...,{a_{{\rm{m}},2}},...,{a_{1,{\rm{n}}}},...,{a_{{\rm{m}},{\rm{n}}}}} \right]^{\rm{T}}}$.

Then we define the proposed transformation formally as follows:

$${\rm vec}_{124,1024}^{ - 1}:{{\rm {\mathbb{R}}}^{1 \times 126,976}} \to {{\rm {\mathbb{R}}}^{124,1024}},$$

$${\rm vec}_{32,32}^{ - 1}:{{\rm {\mathbb{R}}}^{1 \times 1024}} \to {{\rm {\mathbb{R}}}^{32,32}},$$

$${S}^{\prime} = {\rm vec}_{32,32}^{ - 1}\left( {{\rm vec}_{124,1024}^{ - 1}\left( { S} \right)} \right).$$

where $S$ is one patient’s signal data, and $S^{\prime}$ is the patient’s data converted to the square matrix, which can be represented as 2D image.

**Figure 2:**
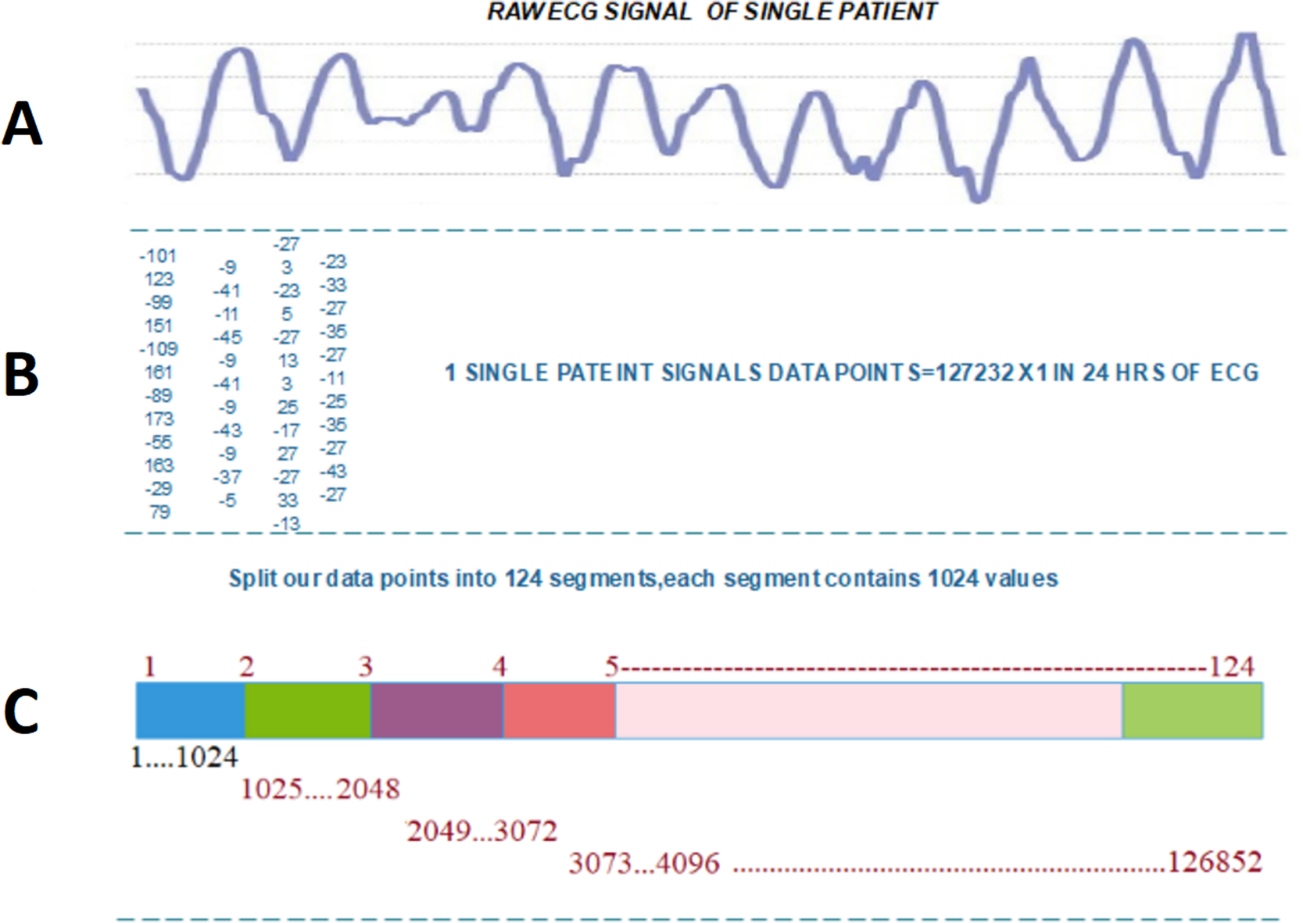
Transforming ECG signal into 124 segments of 1024 values: (A) raw ECG signal, (B) digitized ECG signal, (C) ECG signal segment transformed to image.

Figure 2 illustrated the splitting of an image, which describes each step-in detail on how to transform the ECG signal to a binary image.

**Figure 3:**
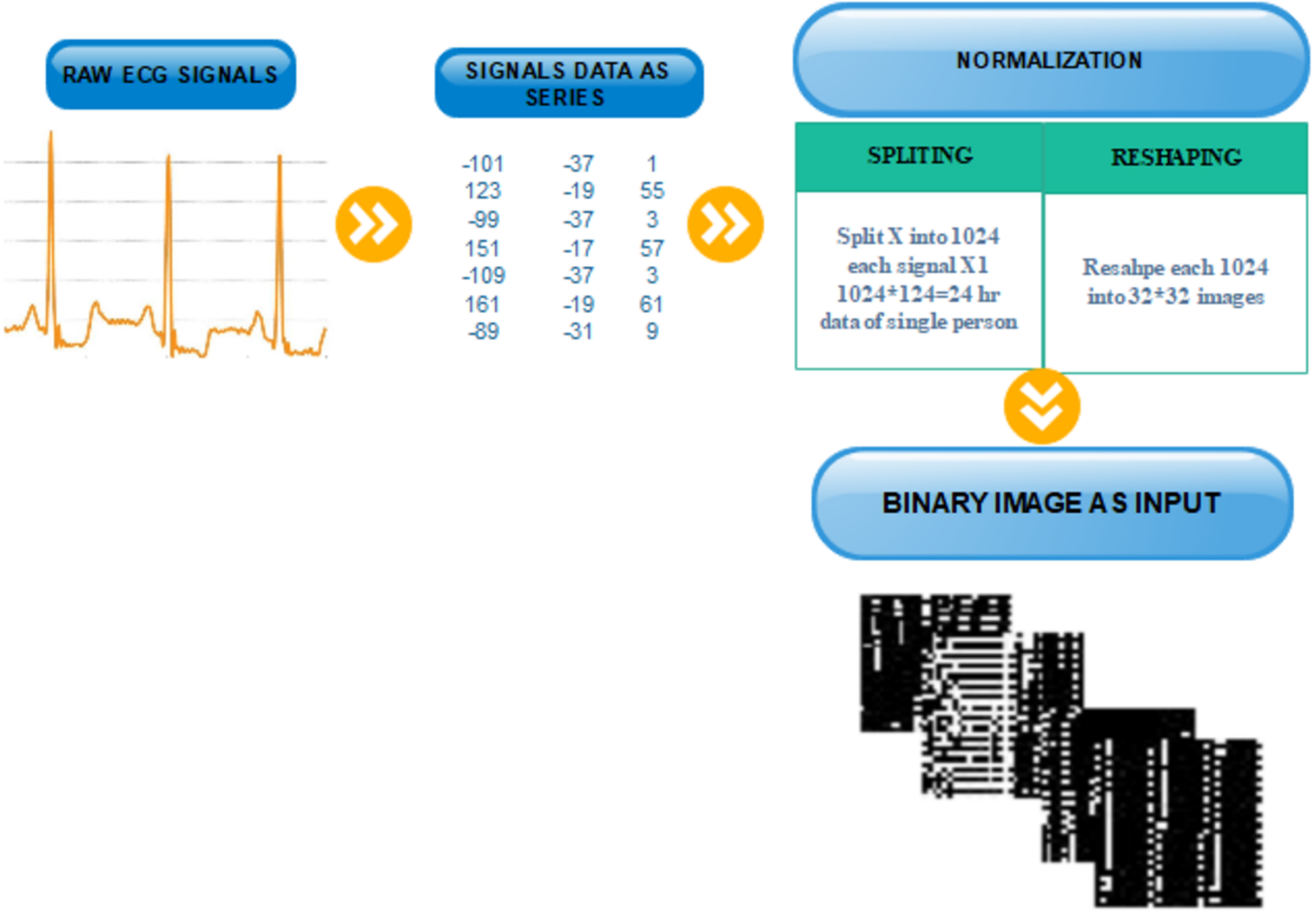
Transformation from one-dimensional signal to a two-dimensional image.

After splitting and reshaping successfully, we save the new dataset. The new dataset contains normal and abnormal images of serial data of signals for multiple patients. The sample images of our new dataset and the detail of transformation are described in Fig. 3.

### Pre-trained CNN features

Deep CNN models have been successfully used for solving numerous tasks in computer vision. CNN takes an input image, forwards it to different layers, for instance, convolutional, nonlinear, fully connected, and pooling to get an output. In computer visualization, transfer learning (TL) is typically expressed using pre-trained models. Because of the high computational cost of training such models from scratch, the pre-trained models can be used.

For feature extraction, we adopt three pre-trained deep CNN models (VGG19, AlexNet and Inception-v3) for deep feature extraction. These models were selected because of their high robustness and proven efficiency in biomedical data ana applications. The purpose of adopting these three models is to process different size images and get depth features. To complete this process, we first resize the $32 \times 32$ image into different sizes $224 \times 224 \times 3$ for AlexNet and VVG19 and $299 \times 299 \times 3$ for Inception-v3. Besides, we convert the binary image ${I_{\rm binary}}$ into three-colour space using the following manner as input for pre-trained models.

$${I_{\rm grayscale}} = \left( {{I_{\rm binary}} \ast 255} \right)$$

$${I_{\rm color}} = {\rm concat}\left( {{I_{\rm grayscale}},{I_{\rm grayscale}},{I_{\rm grayscale}}} \right)$$

**Figure 4:**
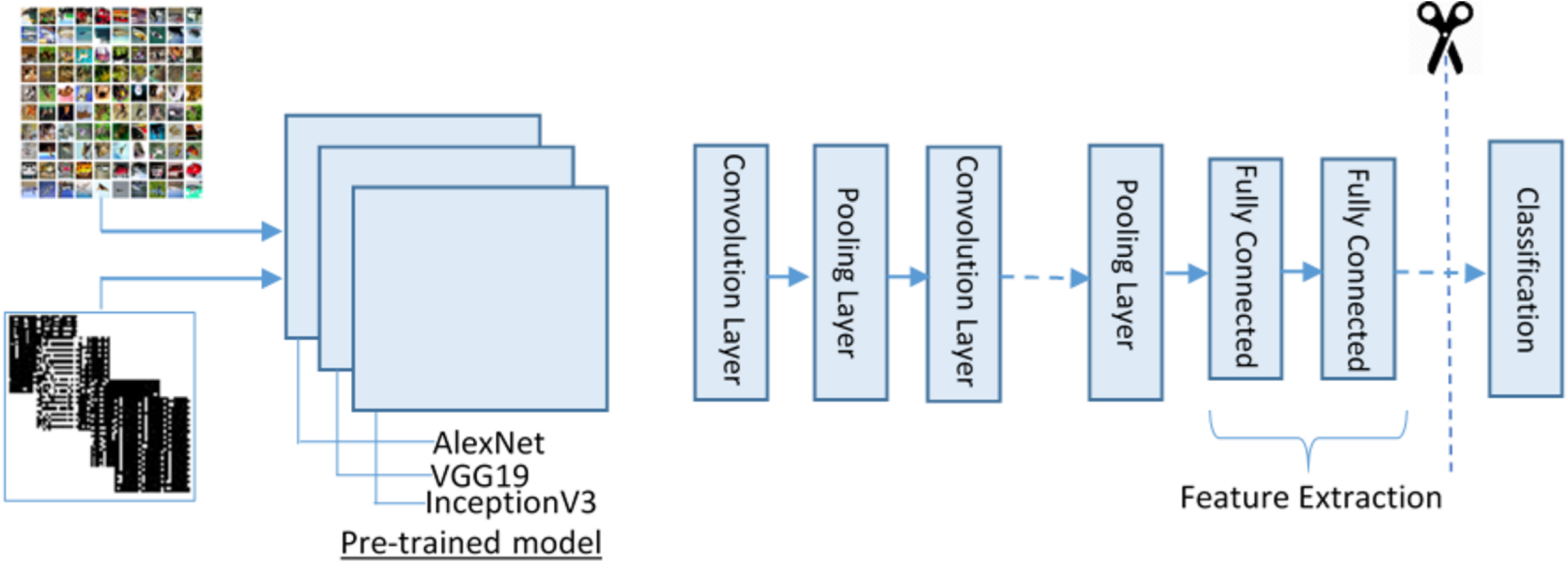
The transfer learning model for feature extraction.

The proposed feature extraction using transfer learning (TL) is illustrated in Fig. 4. TL is described as the potential of a machine to use knowledge and skills learned while solving one set of problems (source) to a different set of problems (target). The purpose of the TL is to improve the performance of a new dataset based on the existing model and to acquire useful features and classification. It can be described mathematically as:

$${D_{s}} = \left\{ {I_1^{\rm s},I_2^{\rm s},I_3^{\rm s},...,I_{\rm n}^{\rm s}} \right\} \to \left\{ {I_1^{\rm T},I_2^{\rm T},I_3^{\rm T},...,I_{\rm n}^{\rm T}} \right\}$$

Where $I$ represents an image, $S$ and $T$ represent labels of training data of source and target domain.

The training on images is done by using DCNN pre-trained models and get 1 × 4,096 features from AlexNet using FC7 layer and 1 × 4,096 features from VGG19 using FC7 layer and 1 × 2,048 features from Inception-v3 using avg-pool known as ${f_1},{f_2},{f_3}$, respectively.

Feature fusion is performed by concatenating features from three neural networks. We adopted an approach similar to the one proposed in Ma, Mu & Sha (2019). The concatenation is performed as follows:

$${F_{v}} = \left[ {{f_1},{f_2},{f_3}} \right]$$

Therefore, the concatenation process is enriching feature diversity to make the classifier perform better. Afterward, these features are fused, concatenated up, and finally get 10,240 features from these models. Here, FC represents a fully connected layer and follows the same structures of the connected feed-forward network, and it can be defined as:

$${F_{v}} = {\rm sig}\left( {\mathop \sum \limits_{i = 1}^{n} {x_{i}} \times w_{\rm i}^{\rm T} + b} \right)$$

where ${x_{i}}$ is known as an input vector of i-th class, $w$ and $b$ represent the weight and bias of constant value.

After fusion, features are fed further for classification. By the reduction of the features, the execution time is decreased with increased performance. Here, the entropy-based feature reduction method is used, which can diminish the number of features based on entropy value. We compute the entropy of fused features using the following equation:

${\rm EE}\left( {{F_{v}}} \right) = {p_{i}}\mathop \sum \nolimits_{\rm i}^{\rm n} {F_\rm v}$,

where (${F_{v}} \in {\rm \; }{f_{\rm 1}},{\rm \; }{f_{\rm 2}}\;{\rm and}\;{f_{\rm 3}}$), and ${p_\rm i}$ is the probability of the extracted feature space, which is defined by$\; Pi{\rm \; } = {\rm \; }pr\left( {X{\rm \; } = i} \right)$ and denotes the size of all feature spaces, which gives a new reduced feature $1 \times 5,120$ feature vector which is 50% of the total features and fed these features to the classifier.

**Figure 5:**
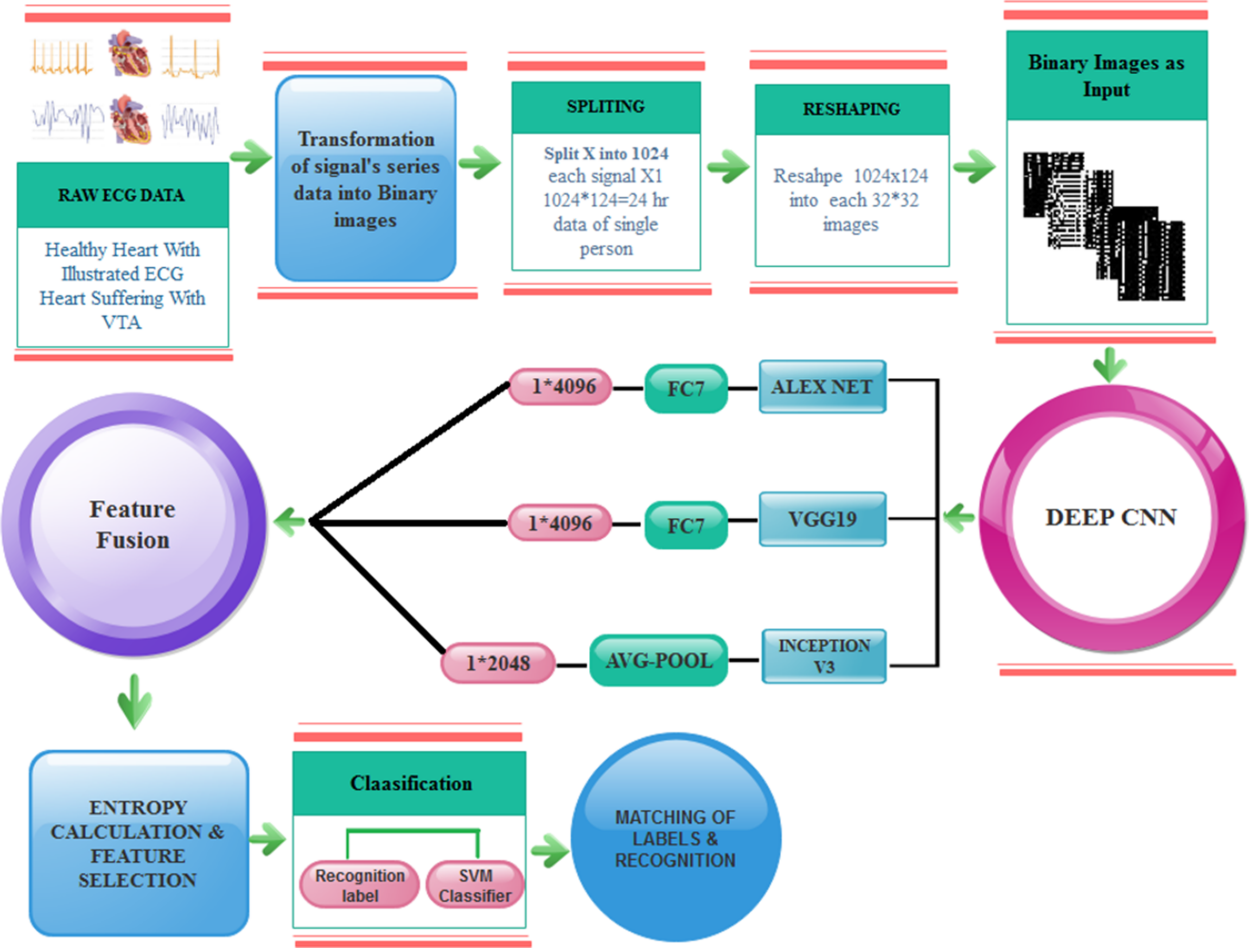
Proposed model for VTA detection.

The overall model of the proposed fast VTA detection is depicted in Fig. 5.

## Results and analysis

For performance measure, the following metrics are determined, where true positive represents correctly recognized VTA, false positive shows incorrectly recognized VTA and false negative determines inaccurately rejected VTA. Results of the proposed method are computed using five different experiments. In the first experiment, DCNN features are extracted using the AlexNet model by performed activations on the Fully Connected layer FC7. The data division approach of 50:50 is adopted for training and testing to validate the proposed technique. Also, the 10-fold cross-validation is adopted on all experimental results. During this experiment, the best testing classification accuracy for AlexNet is recorded at accuracy 91.2%, FNR 8.0%, sensitivity of 91.9%, and specificity 90.5% while using Cubic SVM as final stage classifier. Results after classification on Cubic SVM are crosschecked with nine other classifiers, as it is shown in Table 2.

**Table 2:** Classification results on AlexNet DCNN features. Best values are shown in bold.

Classifier	Performance measures				
	Sensitivity (%)	Specificity (%)	FNR (%)	Accuracy (%)	*F*-score
Cubic SVM	**91.9**	**90.5**	**8.0**	**91.2**	**0.913**
Linear discriminant	72.6	69.8	27.3	71.2	0.716
Linear SVM	86.1	85.8	12.6	86.4	0.860
Quadratic SVM	91.2	90.1	8.7	90.7	0.907
Fine KNN	89.3	86.9	10.6	88.1	0.882
Medium KNN	92.9	83.8	7.0	88.4	0.889
Cubic KNN	93.1	83.6	6.8	88.4	0.889
Weighted KNN	91.9	85.6	8.0	88.9	0.891
Subspace discriminant	74.1	66.4	25.8	70.3	0.714
Subspace KNN	89.7	87.2	10.2	88.5	0.886

In the second experiment, the deep CNN features are extracted using the VGG19 model by performed activations on the Fully Connected layer FC7. Similarly, with AlexNet, a data division approach of 50:50 is adopted for training and testing to validate the proposed technique. Moreover, the 10-fold cross-validation is adopted on all experimental results. During this experiment, the best testing classification accuracy for VGG19 was recorded at 92.1%, FNR 7%, sensitivity of 93.0% and specificity 92.0% using quadratic SVM as a classifier. The results after classification with quadratic SVM are crosschecked with eight other classifiers as it is depicted in Table 3.

**Table 3:** Classification results on VGG19 DCNN features. Best values are shown in bold.

Classifier	Performance measures				
	Sensitivity (%)	Specificity (%)	FNR (%)	Accuracy (%)	*F*-score
Quadratic SVM	**93.0**	**92.0**	**7**	**92.1**	**0.925**
Linear SVM	88.8	88.1	11.11	88.2	0.885
Cubic SVM	92.0	92.0	8	91.9	0.920
Fine KNN	87.5	90.6	12.5	89.2	0.889
Medium KNN	93.0	91.6	7	90.4	0.924
Cubic KNN	93.0	91.6	7	90.4	0.924
Weighted KNN	91.0	91.0	9	90.9	0.910
Subspace discriminant	90.3	90.1	9.6	90.2	0.902
Ensemble subspace KNN	88.3	91.5	11.6	90.0	0.897

Furthermore, the next experiment on DCNN features extracted using the InceptionV3 model by performing activations on the Avg-Pool layer. For this purpose, the same data division approach of 50:50 is adopted for training and testing to validate the proposed technique. The 10-fold cross-validation is adopted on all experimental results. During this experiment, the best testing classification accuracy for InceptionV3 was recorded at 91.5%, FNR 7.7%, sensitivity of 92.2% and specificity 90.9% with Quadratic SVM. Results after classification on Quadratic SVM are crosschecked with seven other classifiers in Table 4.

**Table 4:** Classification results on VGG19 DCNN features. Best values are shown in bold.

Classifier	Performance measures				
	Sensitivity (%)	Specificity (%)	FNR (%)	Accuracy (%)	*F*-score
Quadratic SVM	92.2	90.8	7.7	91.5	0.916
Linear SVM	90.8	87.6	9.1	89.2	0.894
Cubic SVM	**93.4**	**91.6**	**6.6**	**92.5**	**0.926**
Fine KNN	88.1	90.6	11.8	89.4	0.892
Medium KNN	92.2	89.2	7.7	90.7	0.908
Cubic KNN	92.4	87.7	7.5	90.1	0.903
Weighted KNN	90.8	91.3	9.1	91.1	0.910
Ensemble subspace discriminant	89.4	89.3	10.5	89.4	0.894

In the next experiment, fusing the features obtained from AlexNet, VGG19, and InceptionV3 is performed. These feature vectors are fused to make a standalone feature vector representing all three pre-trained models. 10-fold cross-validation is adopted on all experimental results. During this experiment, the best testing classification accuracy for fused feature vector was recorded at 96.6%, FNR 3.0%, sensitivity of 97.12% and specificity of 95.99% on Cubic SVM. Results after classification with Cubic SVM are crosschecked with six other classifiers in Table 5.

**Table 5:** Classification results after performing the fusion of the AlexNet, VGG19, Inceptionv3 DCNN features. Best values are shown in bold.

Classifier	Performance measures				
	Sensitivity (%)	Specificity (%)	FNR (%)	Accuracy (%)	*F*-score
Cubic SVM	**97.12**	**95.99**	**3.0**	**96.6**	**0.966**
Linear SVM	95.53	91.48	4.56	93.5	0.936
Quadratic SVM	96.68	95.51	3.3	96.1	0.961
Fine KNN	93.81	92.4	6.1	93.1	0.932
Medium KNN	97.40	89.15	2.59	93.3	0.935
Cubic KNN	96.86	88.35	3.13	92.6	0.929
Weighted KNN	96.23	91.48	3.76	93.9	0.940

In another experiment, we performed entropy-based feature selection. We calculated the entropy of fused features. After entropy calculation, we choose entropy-based features from it and start from the first 1,000 features and train them. In the second step, we take the first 2,000 and up to 8,000 features. The entropy of the fused feature vector is calculated, which derived an entropy feature vector. Therefore, the entropy vector is sorted into ascending order. We get the highest accuracy on selecting 5,120 features, which are 50% of our data. After that, we take 25% of the data, but the accuracy decreases.

**Figure 6:**
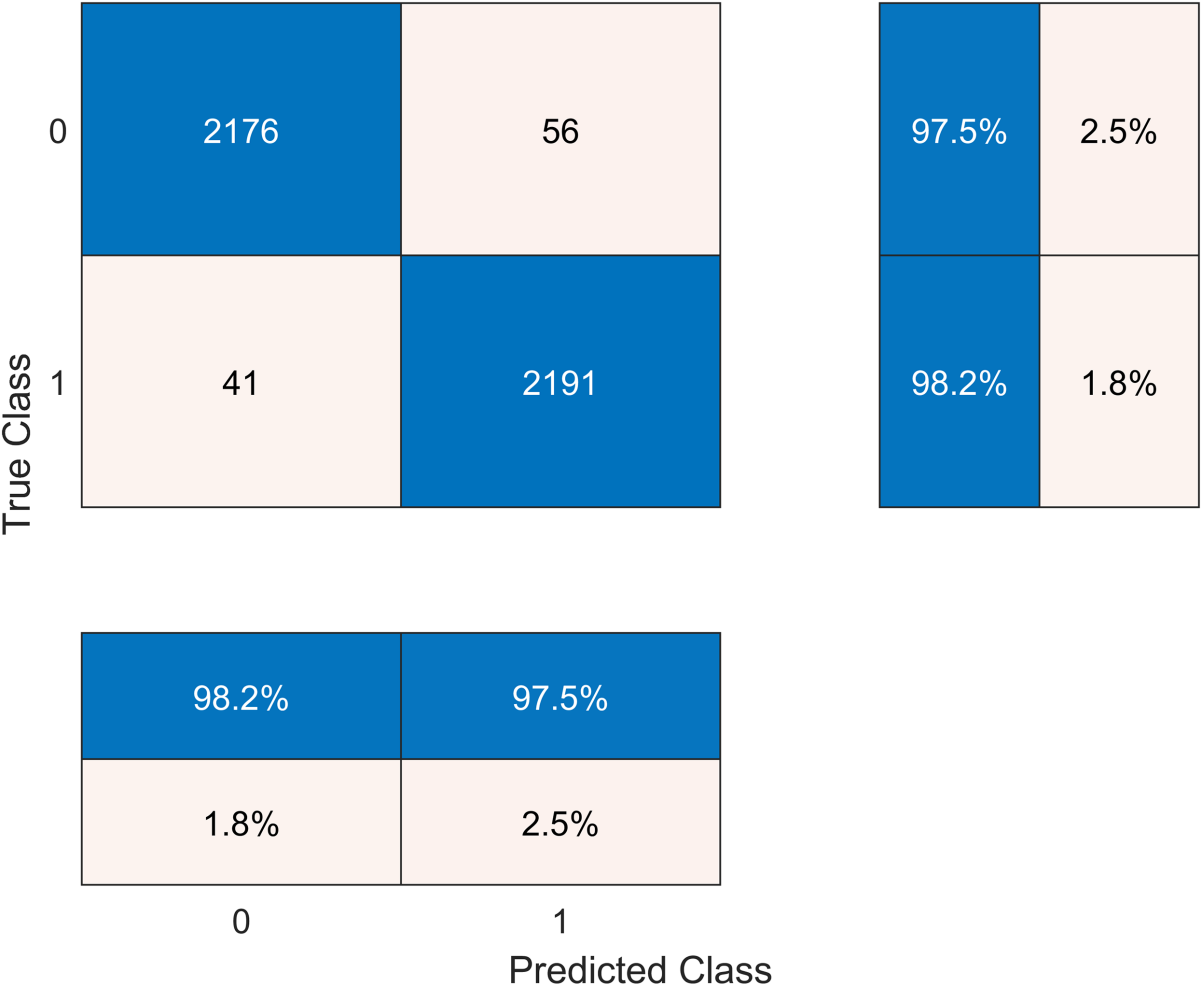
Confusion matrix of the best performing model.

The results after classification on the Cubic SVM are crosschecked with seven other classifiers in Table 6. We present the confusion matrix of the best accuracy model in Fig. 6. Besides, we have experimented on the Cubic SVM by selecting the features in ascending order on 10-fold cross-validation, as it is shown in Table 7.

**Table 6:** Classification results on the fused Alexnet, VGG19, Inceptionv3 DCNN features after entropy based selection. Best values are shown in bold.

Classifier	Performance measures				
	Sensitivity (%)	Specificity (%)	FNR (%)	Accuracy (%)	*F*-score
Cubic SVM	**98.2**	**97.5**	**2.3**	**97.6**	**0.979**
Linear SVM	95.16	91.7	4.8	93.5	0.935
Quadratic SVM	96.50	95.5	3.4	96.0	0.960
Fine KNN	93.5	94.0	6.4	93.8	0.937
Medium KNN	96.7	90.68	3.1	93.8	0.939
Cubic KNN	96.41	89.6	3.5	93.1	0.932
Weighted KNN	96.31	89.2	3.3	93.1	0.930

**Table 7:** Accuracy of classification on different selected sets of features.

Numbers of features	Accuracy (%)	Training time (s)
1,000	93.5	29.609
2,000	94.0	33.149
3,000	95.9	66.200
4,000	96.2	85.856
5,000	97.0	109.880
5,120 (50%)	97.6	111.690
2,560 (25%)	95.7	55.151
6,000	96.9	144.810
7,000	96.9	166.360
8,000	96.6	196.040

### Comparison with other works

There are most of the traditional approaches and latest techniques like CNN and deep learning for the diagnosis of VTA. However, from the literature, this can be well known that these computer-aided diagnostic (CAD) systems have a consistent workflow. For example, one system achieved an accuracy of 94.07% and 91.5% on the MIT-BIH dataset (Acharya et al., 2017); moreover, they are also time-consuming. The latest technique, which included a deep CNN to make the algorithm automated, gained a higher accuracy of 97.6% on a similar dataset (Ullah et al., 2020). Ullah et al. (2020) transformed EEG signals into 2-D spectrograms through short-time Fourier transform. Then they used the 2-D CNN model consisting of four convolutional layers and four pooling layers for classification of ECG signals into eight classes, achieving the average accuracy of 99.11%. Jin et al. (2020) proposed the Domain Adaptive Residual Network (DARN) to detect Afib from ECG signals, achieving 77.97% accuracy on the MIT-BIH Arrhythmia Database. Li et al. (2020) suggested CraftNet, a custom deep neural network with tailored architecture, which achieved an average sensitivity of 89.25% on the MIT-BIH dataset. Romdhane et al. (2020) suggested another CNN architecture, which achieved 98.41% average accuracy. Van Steenkiste, Van Loon & Crevecoeur (2020) proposed a deep neural network with a parallel convolutional neural network architecture, optimized by a Genetic Algorithm (GA), for ECG beat classification, an achieved an accuracy of 97.7% on the MIT-BIH dataset. Yang et al. (2021) proposed an ensemble multiclass classifier that combined mixed-kernel-based extreme learning machine (MKELM) as base learner and random forest as a meta-learner, achieving an overall accuracy of 98.1% in classifying four types of heartbeats.

The findings of the literature confirm our finding that if we transform our signals data into images then CNN works better, and we get the highest accuracy using different classifiers. We summarize the related works in Table 8.

**Table 8:** Comparison of results achieved on the MIT-BIH dataset.

Reference	Methods	Accuracy (%)
Acharya et al. (2017)	Custom 9-layer deep CNN	94.03
Ullah et al. (2020)	2D CNN model applied on FFT spectrograms	99.11
Jin et al. (2020)	Domain Adaptive Residual Network	
Li et al. (2020)	Fully-connected neural networks as classifiers on handcraft features	89.25
Romdhane et al. (2020)	Custom 1D CNN	98.41
Van Steenkiste, Van Loon & Crevecoeur (2020)	Custom CNN optimized by Genetic Algorithm (GA)	97.7
Yang et al. (2021)	Ensemble of mixed-kernel extreme learning machine-based random forest binary classifiers	98.1
This article	Deep features from 3 deep networks and Cubic SVM	97.6

## Discussion and conclusions

There are many the traditional approaches and latest techniques like CNN and deep learning used for the diagnosis of VTA. The main problem occurs when different cofactors affect like QRS complex and segmentation. If the data is not appropriately segmented, the accuracy problem occurs in the prediction of VTA. The significant problems that occur after applying the pattern recognition technique are:

- the amount of data came out for processing is enormous, it is difficult to manage and process those large amounts of values;
- the limitations of traditional techniques and methods—the previous techniques are restricted to single feature searching capabilities of signals;
- cardiac cycle dynamics reflect underlying physiological changes.
- big data is required for training, which takes more time.

From the analysis of related literature, it concludes that there are recent surveys that involved CNN for the prediction of arrhythmias but the highest accuracy they achieved is 91.2%. CNN models are not working well on signals data as mentioned in the problem statement. For this problem we need to convert one-dimensional signal data into a two-dimensional image (matrix). This is a big challenge here to normalize signal data and transform it into binary image without loss of any information, because ECG signal are non-stationary.

To overcome the above problems, we introduced a novel approach where the main contribution is to convert ECG signals into binary images and automate VTA detection using deep learning and get higher accuracy and less time consumption. The proposed model is tested on MIT/BIH using pre-trained models, Alex Net, VGG19, and InceptionV3. Higher accuracy (97.6% using Cubic SVM as a final stage classifier) is achieved than existing methods, and the execution time is minimized too by making the algorithm automated using CNN.

### Future work

This study leads to a future direction where the aim to make a variant architecture of the network model for the prediction of different arrhythmias, including ventricular and atrial. In future work, the framework will be trained and tested on big data. If this processing of feature fusion and feature selection can be applied to other domains after selecting the required features, the results might improve performance in terms of effectiveness and efficiency. The proposed technique is not only limited to the ECG image classification. It can be applied to any other domain such as electroencephalography (EEG), which is directly connected with efficient feature extraction, fusion and selection.

## Supplemental Information

10.7717/peerj-cs.386/supp-1 Supplemental Information 1 Code of method implementation (MATLAB).

## Additional Information and Declarations

### Data Availability

The following information was supplied regarding data availability: Data is available at PhysioNet: DOI 10.13026/C2F305 MATLAB code is available in a Supplemental File.
